# AoRan1 Is Involved in Regulating Conidiation, Stress Resistance, Secondary Metabolism, and Pathogenicity in *Arthrobotrys oligospora*

**DOI:** 10.3390/microorganisms12091853

**Published:** 2024-09-06

**Authors:** Shipeng Duan, Qianqian Liu, Yanmei Shen, Lirong Zhu, Hui Yuan, Jinkui Yang

**Affiliations:** State Key Laboratory for Conservation and Utilization of Bio-Resources in Yunnan, Key Laboratory for Southwest Microbial Diversity of the Ministry of Education, and School of Life Science, Yunnan University, Kunming 650032, China; duanshipeng@stu.ynu.edu.cn (S.D.); liuqianqian614@163.com (Q.L.); shenyanmei@stu.ynu.edu.cn (Y.S.); zhulirong_dif5@stu.ynu.edu.cn (L.Z.); yuanhui201228@163.com (H.Y.)

**Keywords:** serine/threonine protein kinase, sporulation, lipid droplet, autophagy, secondary metabolite, nematode-trapping ability

## Abstract

*Arthrobotrys oligospora* is a representative nematode-trapping (NT) fungus that is able to capture, kill, and digest nematodes by producing specialized three-dimensional networks (traps) under nutrient-deprived conditions. Ran1 is a serine/threonine protein kinase that can act as a negative regulator of sexual conjugation and meiosis. However, the specific role of Ran1 remains largely unknown in NT fungi. Here, we identified AoRan1 (AOL_s00004g277) via gene disruption, phenotypic analysis, and metabolomic analysis. Our findings reveal that *Aoran1* knockout caused a remarkable increase in conidial production, traps, and nematode feeding efficiency. In addition, the absence of *Aoran1* resulted in the accumulation of lipid droplets and increased autophagic levels as well as increased tolerance to cell wall synthesis-disturbing reagents and oxidants. Metabolomic analyses also suggested that AoRan1 is involved in multiple metabolic processes, such as fatty acid biosynthesis. In summary, our results suggest that AoRan1 is crucial in conidiation, pathogenicity, and secondary metabolism. This study’s results further our understanding of the molecular mechanisms by which AoRan1 regulates conidiation and trap formation in *A. oligospora*.

## 1. Introduction

Plant parasitic nematodes (PPNs) are plant pathogens that cause a significant impact on annual global crop productivity [1,2]. Current control measures primarily rely on chemical nematicides, but there is growing concern about their environmental impact [3]. Therefore, there is an urgent need to find an efficient and non-polluting nematode control measure [4]. Nematode-trapping (NT) fungi are promising potential biological control agents against PPNs due to their ability to trap, kill, and consume nematodes by producing diverse trapping devices (traps), including constricting rings and adhesive traps (adhesive knobs, adhesive nets, adhesive columns, and non-constricting rings) [5,6,7,8,9]. Most NT fungi live saprophytically, but once nematodes are present, their vegetative hyphae differentiate into traps to capture the nematodes as a supplement [10]; this also represents a shift in NT fungi towards a predatory lifestyle [11,12]. As a representative NT fungus, *A. oligospora* is capable of producing three-dimensional networks to consume nematodes; because of this, it is widely used as a model strain to explore fungal interactions with nematodes [11,13]. The number and morphology of traps are critical for the nematode predation efficiency of NT fungi; thus, the molecular mechanisms of the signaling pathways associated with trap formation are becoming a focus of research.

Studies have shown that protein kinases are the largest family of signal transduction proteins in eukaryotic cells, and the majority of them are serine/threonine protein kinases [14,15,16]. The phosphorylation of serine/threonine protein kinases has been proven to be involved in regulating various biological pathways, such as metabolism, cytoskeletal reassembly, mitotic cell division, and the regulation of membrane receptors [14,17,18,19]. Ran1 is a conserved serine/threonine protein kinase and a negative regulator of sex conjugation and meiosis, and its function is well understood in the fission yeast *Schizosaccharomyces pombe* [20,21,22]. For instance, when nutritional deficiencies occur, cells end the mitotic process and instead undergo conjugation and meiosis, and Ran1 plays an important role in this process. Ran1 kinase functions as a negative regulator of Mei2, an RNA-binding protein that is essential for meiotic initiation. The inactivation of Ran1 is followed by the activation of Mei2, which initiates meiosis under nutrient-deficient conditions via a signaling cascade involving Ste11 [22,23,24,25]. The Ste11 protein is also a component of the pheromone-responsive mitogen-activated protein kinase (MAPK) cascade containing the MAPK Fus3 protein [26]. Previous studies have also shown that the MAPK pathway is phosphorylation-dependent and activated by different protein kinases in which the serine/threonine protein kinases play an important role [27,28]. A study demonstrated that the deletion of Ran1 significantly affects the transcription levels of genes regulating the MAPK signaling pathway [29]. The MAPK signaling pathway has been found and proven to regulate multiple biological processes in filamentous fungi, including NT fungi [30,31]. These results suggest that Ran1 may be associated with the MAPK signaling pathway. However, the specific role of Ran1 remains largely unknown in NT fungi. Considering the above results, we speculate that Ran1 plays a key regulatory role in multiple biological processes in *A. oligospora*, such as conidiation and nematode-trapping ability.

Here, we investigated the roles of the *Aoran1* gene via multiple phenotypic and metabolomic analyses. Our results reveal that AoRan1 plays a crucial role in conidiation, trap formation, lipid droplet (LD) accumulation, the autophagic level, pathogenicity, and secondary metabolism.

## 2. Materials and Methods

### 2.1. Strains and Culture Conditions

The wild-type (WT) *A. oligospora* (ATCC24927), obtained from the Microbial Library of the Germplasm Bank of Wild Species from Southwest China, and the WT and deletion strains used in this study were incubated routinely in potato dextrose agar (PDA; 200 g potato, 20 g dextrose, and 20 g agar per 1 L) medium under dark conditions. The pCSN44 plasmid used to amplify the hph cassette and pRS426 plasmid for the construction of knockout vectors are conserved in DH5α (TaKaRa, Dalian, China). *Saccharomyces cerevisiae* FY834 was cultured on yeast extract peptone dextrose (YPD; 10 g yeast extract, 20 g peptone, 20 g dextrose, and 20 g agar per 1 L) medium. The Caenorhabditis elegans N2 strain was cultured in oatmeal medium and used to induce trap formation.

### 2.2. Phylogenetic Analysis of AoRan1

On the basis of the amino acid sequence of homologous Ran1 encoded by the model fungi *Aspergillus fumigatus* and *Neurospora crassa*, the AoRan1 protein was identified in *A. oligospora* through comparative analysis using the NCBI database. The isoelectric point (PI) and molecular weight (MW) of AoRan1 were calculated using ExPASy-ProtParam (http://web.expasy.org/compute_pi/) (accessed on 5 July 2024). The conserved functional domains were predicted using InterProScan (http://www.ebi.ac.uk/Tools/pfa/iprscan/) (accessed on 5 July 2024). On the basis of the amino acid sequences of Ran1 homologs in diverse fungi, a phylogenetic tree was built using MEGA 7.0 [32].

### 2.3. Deletion of Aoran1

The target gene *Aoran1* (AOL_s00004g277) was deleted by means of homologous recombination [33]. In brief, the 5′ and 3′ flanking regions of the *Aoran1* open reading frame were amplified from *A. oligospora* genomic DNA using the designed primers; the *hph* fragment with hygromycin resistance was amplified from the pCSN44 plasmid and used as a screening marker. The linearized plasmid pRS426 (*Eco*RI and *Xho*I*)* was co-transduced into *S. cerevisiae* FY834 with the amplified fragments to construct a knockout vector for the target gene. The plasmids we used in this study are shown in Figure S1. Eventually, the correctly constructed knockout vector was inserted into the protoplasts via PEG-mediated transformation [34]. Then, transformants were screened on a PDAS (PDA supplemented with 10 g/L molasses and 0.4 M saccharose) medium with 200 µg/mL of hygromycin B (Amresco, Solon, OH, USA) [35] and validated using the PCR and quantitative real-time PCR (RT-qPCR) methods. RT-qPCR was applied to detect the transcription level of the target gene in positive transformants. The primers used for the knockout of the target gene and validation of the transformants are shown in Table S1.

### 2.4. Comparison of Hyphal Growth and Sporulation

The growth rates of the fungal strains under diverse nutritional circumstances were compared by removing discs of the same size from the colony edge after 5 days of activation on PDA and inoculating them into three different media, PDA, TG (10 g tryptone, 10 g glucose, and 20 g agar per 1 L), and TYGA (10 g tryptone, 5 g yeast extract, 10 g dextrose, 5 g molasses, and 20 g agar per 1 L), respectively. After incubation for 5 days at 28 °C under dark conditions, the colony diameter was measured and recorded every 24 h [30]. To calculate the production of conidia, the mycelia were eluted and filtered with 10 mL ddH_2_O from the fungal strains incubated in corn meal yeast extract (CMY, 20 g corn starch, 5 g yeast extract, and 20 g agar per 1 L) medium. Then, the spore suspension in 1 μL was examined using a microscope as previously described [36,37]. The number of conidia in each sample was counted three times.

### 2.5. Comparison of Mycelial Growth under Different External Pressures

To explore the ability of AoRan1 to respond to external stressors, the fungal strains were incubated in TG solid medium with different concentration gradients of chemical stress reagents, including oxidants (H_2_O_2_ and menadione), osmotic stressors (NaCl and sorbitol), and cell wall synthesis-disturbing reagents (SDS and Congo red) for 5 days under dark conditions. TG medium without any added reagents was used as a control. The relative growth inhibition (RGI) values were calculated as previously described [34,37]. Three biological replications were performed for each sample.

### 2.6. Comparison of Pathogenicity and Observation of Trap Morphology

To compare the capacity to produce traps between the WT and ∆Aoran1 mutant strains, approximately 20,000 spores eluted from the CMY medium were spread evenly in water agar (WA, 20 g agar per 1 L deionized water) medium. Approximately 400 nematodes (*C. elegans* N2) were transferred into the medium to induce trap formation [38]. The nematode capture rate was observed, and the number of traps was counted every 12 h up to 48 h. The morphology of mature traps across the fungal strains was observed by means of calcofluor white (CFW) staining. In addition, discs of the same size were removed from the colony edge of the fungal strains incubated in PDA medium and then incubated in potato dextrose broth for two days in a shaker flask at 180 rpm. To compare the size of the protease hydrolysis circles, the same amounts of fermentation broth from the fungal strains were dropped into wells punched out of a WA plate containing skim milk [39].

### 2.7. Staining Analysis

The fungal strains were cultured in PDA medium with coverslips inserted into the medium to allow mycelia to attach to them. For the staining of septa and nuclei, the mycelia were stained with 20 μg/mL CFW (Sigma-Aldrich, St. Louis, MO, USA) and 20 μg/mL 4′, 6-diamidino-2-phenylindole (DAPI) (Sigma-Aldrich). In order to observe differences in fatty acid metabolism and autophagic levels, the mycelia of the fungal strains were stained with 10 µg/mL of boron dipyrromethene (BODIPY) dye (Sigma-Aldrich) and 10 µg/mL of monodansylcadaverine (MDC, Sigma-Aldrich), respectively. The staining time was approximately 5–10 min. All of the above staining results were visualized using an inverted fluorescence microscope (ECLIPSE Ni-E, Nikon, Tokyo, Japan) [36,40]. In addition, transmission electron microscopy (TEM) (JEM-1400Plus, Hitachi, Tokyo, Japan) was applied to observe the ultrastructure of hyphal cells.

### 2.8. RT-qPCR Analysis

The fungal strains were inoculated in PDA medium covered with a cellophane membrane, and mycelia were collected on day 5. The RNA of the mycelial samples was extracted with an RNA extraction kit (Axygen Scientific, Union City, CA, USA). Then, the extracted RNA was reversely transcribed into stable cDNA using a PrimeScript RT Reagent Kit with a gDNA Eraser (TaKaRa, Dalian, China). The β-tubulin gene (AOL_s00076g640) was used as the internal standard. The primers used to detect the transcription levels of genes related to sporulation, serine protease, fatty acid metabolism, and the autophagic process are shown in Table S2. The transcription levels of the genes examined in this study were obtained using the 2^−ΔΔCT^ method [41].

### 2.9. Metabolomics Analysis

The fungal strains were incubated in PDA medium for 5 days and then inoculated into 250 mL of PD broth (200 g potato, and 20 g dextrose per 1 L) and cultured in shake flasks for 7 days. Then, 250 mL of ethyl acetate was mixed with the fermentation broth and ultrasonically shaken for 20 min; shaking was repeated three times. The mixed solution was then left for approximately 12 h. The supernatant solution was extracted using a vacuum rotary evaporator, and then the extracted crude extract was dissolved in 1.5 mL of methanol (chromatographic grade ≥99%). The solution was filtered into a light-protected bottle. Meanwhile, the fermentation broth was filtered through a vacuum pump to collect the mycelia, and the collected mycelia were dried and weighed. Finally, the extracted individual samples were quantified based on their dry weights and then subjected to liquid chromatography–mass spectrometry (LC-MS) analysis. Thermo Xcalibur software (Version 3.0, Thermo Fisher Scientific, Waltham, MA, USA) and Discoverer software 3.0 were applied to analyze the metabolic profiles and perform non-targeted metabolomics, respectively [42].

### 2.10. Statistical Analysis

To make the experimental results more precise, three replications of each experiment were performed. The experimental data are expressed as the mean ± standard deviation (SD), and significance was defined at *p* < 0.05. GraphPad Prism Software (version 9) was applied to perform analyses on the experimental data.

## 3. Results

### 3.1. Sequence Analysis of AoRan1

**Figure 1:**
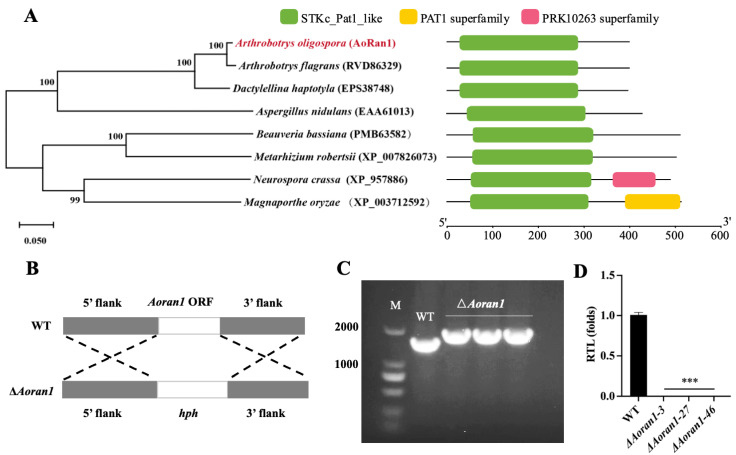
A phylogenetic analysis of AoRan1 and the deletion and verification of the *Aoran1* gene in *A. oligospora*. (**A**) A neighbor-joining tree of orthologous Ran1 in different fungi, which was built using MEGA7.0 (Left), and a prediction of the functional domains of AoRan1 homologs in diverse fungi (Right). (**B**) A schematic diagram of the homologous recombination strategy used to knock out Aoran1. (**C**) The validation of Δ*Aoran1* transformants using PCR. (**D**) The validation of Δ*Aoran1* transformants using RT-qPCR. RTL: the relative transcription level of gene *Aoran1*. Asterisk: Significantly different compared to the WT strain (*** *p* < 0.001).

AoRan1 contains 399 amino acid residues with an MW of 44.02 kDa and a PI of 6.41. The phylogenetic tree shows that AoRan1 shares very high sequence similarity (89.47–97.99%) with the homologous proteins of the other two NT fungi (*Arthrobotrys flagrans* and *Dactylellina haptotyla*), and they also belong to the same branch in the phylogenetic tree. In addition, AoRan1 shares a moderate degree of similarity (62.68–66.67%) with the homologs of certain filamentous fungi, including *Aspergillus nidulans*, *Beauveria bassiana*, *Metarhizium robertsii*, *Neurospora crassa*, and *Magnaporthe oryzae.* The major functional domain of AoRan1 and its homologous proteins is the STKc_Pat1_like domain, which catalyzes the transfer of γ-phosphoryl groups from ATP to serine/threonine residues on the protein substrate and is highly conserved across fungi (Figure 1A).

### 3.2. Verification of Positive Transformants

To further investigate the function of *Aoran1*, homologous recombination was used to disrupt the target gene by replacing it with an *hph* cassette (Figure 1B). The transformants were verified by means of PCR using primers YZ-*AoRan1*-F and YZ-*AoRan1*-R (Figure 1C). The positive transformants were further verified via RT-qPCR to check the transcription level of *AoRan1* (Figure 1D). Then, three positive transformants (Δ*Aoran1–3*, Δ*Aoran1–27*, and Δ*Aoran1–46*) were randomly selected for a series of subsequent experiments.

### 3.3. Comparison of Growth Rates between WT and ∆Aoran1 Mutant Strains

**Figure 2:**
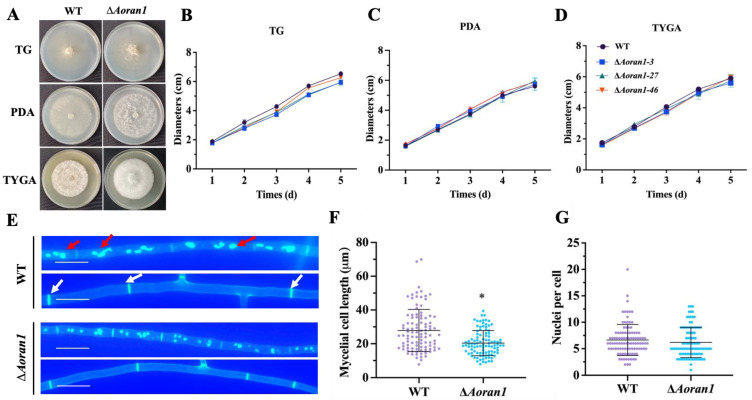
Role of Aoran1 in vegetative growth and mycelial growth. (**A**) Colony morphology after 5 days of incubation in three basal media. (**B**–**D**) Colony diameters in PDA (**A**), TG (**B**), and TYGA (**C**) media. (**E**) Mycelia stained with calcofluor white and 4′, 6-diamidino-2-phenylindole to observe septa and nuclei. Bar: 10 μm. White arrow, hyphal septa; red arrow, nuclei. (**F**,**G**) Statistical analysis of mycelial cell length (**F**) and nuclear number (**G**) in fungal strains. WT, wild-type strain. Asterisk: Significantly different compared to WT strain (* *p* < 0.05).

To explore the impact of ∆*Aoran1* on the growth rate of *A. oligospora*, the fungal strains were incubated in PDA, TG, and TYGA media for 5 days. Compared to the WT strain, the growth rates of the Δ*Aoran1* mutant strains were not obviously different in any of the three media. However, the aerial mycelia of the Δ*Aoran1* mutant strains were denser than those of the WT strain (Figure 2A–D). Meanwhile, CFW staining suggested that the septum number in the Δ*Aoran1* mutant strains was remarkably increased, which caused a remarkable decrease in the mycelial cell length (Figure 2E,F); however, DAPI staining showed no remarkable change in the average number of nuclei (Figure 2E,G).

### 3.4. AoRan1 Plays a Vital Role in Conidiation

**Figure 3:**
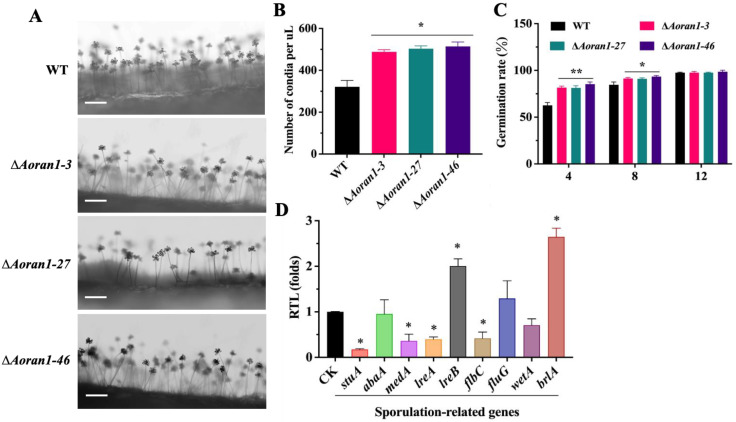
Role of AoRan1 in conidiation. (**A**) Observations of conidiophores in fungal strains. Bar = 100 μm. (**B**) Conidial production in 1 μL conidial suspension, comparing WT and mutant strains. (**C**) Conidial germination rates in fungal strains. (**D**) Relative transcription level (RTL) of several sporulation-related genes in mutant strain versus WT strain, detected using RT-qPCR. CK was denoted as standard for statistical analysis of relative transcription level of gene in mutant versus WT strain. Asterisk: Significantly different compared to WT strain (* *p* < 0.05; ** *p* < 0.01).

In the absence of *Aoran1*, there were no significant differences in the number of conidiophores or the morphology of conidia attached to conidiophores (Figure 3A). However, the absence of Δ*Aoran1* caused a statistically significant increase in the conidial number of *A. oligospora* (Figure 3B). Additionally, the conidial germination rate was significantly increased (Figure 3C). We examined the transcription levels of nine sporulation-related genes by means of RT-qPCR technology; among these genes, the expressions of *lreB* and *brlA* (one of the core regulatory genes of conidiation) were significantly upregulated, whereas the expressions of *stuA*, *medA*, *lreA*, and *flbC* were remarkably downregulated (Figure 3D).

### 3.5. AoRan1 Is Critical to Trap Formation and Pathogenicity

**Figure 4:**
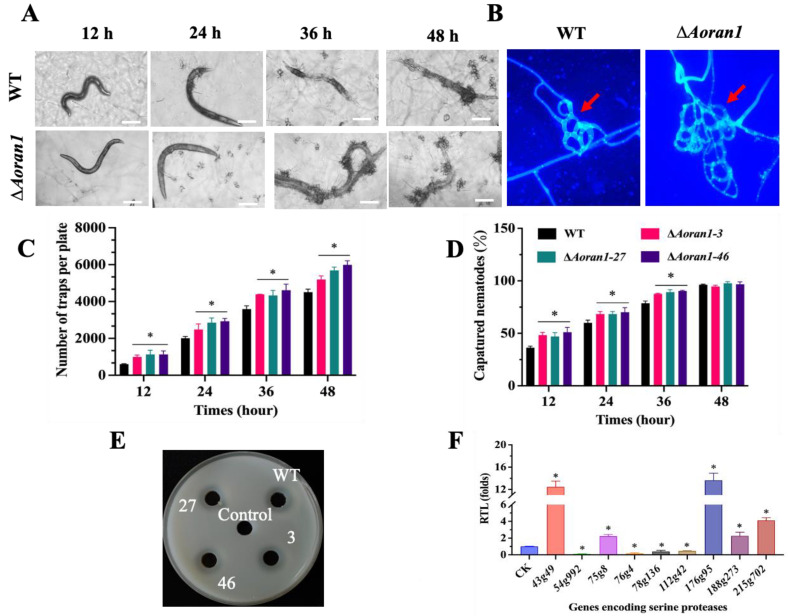
Role of AoRan1 in trap formation, nematode-trapping ability, and proteolytic activity. (**A**) Microscopic images of trap formation and predation on nematodes at 12–48 h. Bar = 100 μm. (**B**) Traps were stained with CFW to observe their morphology. Red arrows: traps. Bar = 20 μm. (**C**) Trap yields of fungal strains at 12–48 h. (**D**) Percentages of nematodes captured by fungal strains at 12–48 h. (**E**) Comparison of hydrolysis circle of protease activity between WT and mutant strains. (**F**) Relative transcription level (RTL) of several genes encoding serine proteases in mutant strains, detected using RT-qPCR. CK was denoted as standard for statistical analysis of relative transcription level of gene in mutant versus WT strain. Asterisk: Significantly different compared to WT strain (* *p* < 0.05).

To further investigate whether *AoRan1* affects trap formation, fresh spores from fungal strains grown on CMY medium were collected; then, approximately 20,000 spores were evenly spread in WA medium and incubated for 4 days. After the addition of approximately 400 nematodes to the medium, trap formation was observed every 12 h (Figure 4A). The morphology of the traps was observed using CFW staining. The mature traps of the WT strain consisted of 5–8 mycelial rings; in contrast, traps in the Δ*Aoran1* mutant strains contained 7–10 mycelial rings, and the conjunction between mycelial rings in the traps was denser, which may indicate a significant increase in hyphal fusion in the traps (Figure 4B). The results show that the deletion of *Aoran1* caused a remarkable increase in the number of traps at all time points (Figure 4C). The nematode-trapping ability was also significantly increased at 12–36 h and then converged to that of the WT strain at 48 h (Figure 4D). Moreover, we compared the proteolytic activity of the Δ*Aoran1* mutant strain with that of the WT strain, and the size of the protease hydrolysis circle did not differ significantly between them (Figure 4E). The transcription levels of nine genes encoding serine proteases were examined via the RT-qPCR method, and the transcription levels of *43g49*, *75g8*, *176g95*, *188g273*, and *215g702* were remarkably upregulated, while those of the remaining genes were remarkably downregulated. In particular, the transcription level of *54g992* was close to zero (Figure 4F). The above results reveal that AoRan1 is crucial in regulating trap formation and virulence.

### 3.6. AoRan1 Is Involved in Stress Response

**Figure 5:**
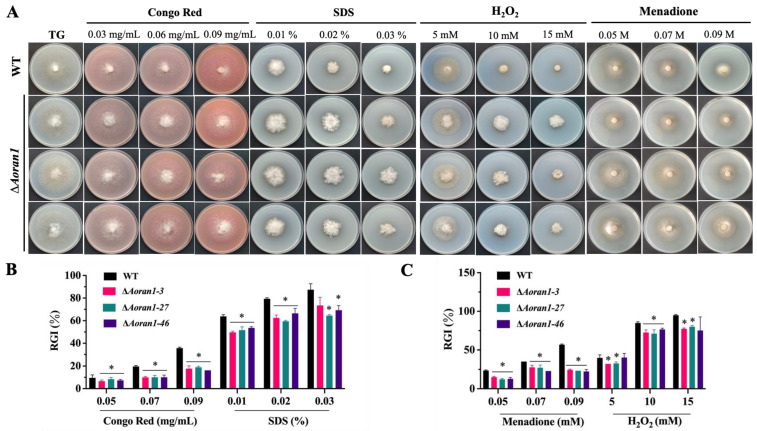
Role of AoRan1 in response to external stress. (**A**) Colonial morphology supplemented with different concentration gradients of cell wall synthesis-disturbing (CWSD) reagents and oxidants. (**B**,**C**) Relative growth inhibition (RGI) rate of fungal strains after 5 days of culture in TG media treated with different concentrations of CWSD reagents (Congo red and SDS) (**B**) and oxidants (H_2_O_2_ and menadione) (**C**). Asterisk: Significantly different compared to WT strain (* *p* < 0.05).

The Δ*Aoran1* mutant strains showed a significant increase in tolerance to different concentration gradients of cell wall synthesis disruptors (Congo red and SDS) and oxidants (H_2_O_2_ and menadione) (Figure 5A), and the deletion of *Aoran1* caused a faster growth rate compared to the WT strain, with a corresponding significant decrease in RGI values (Figure 5B,C). Compared with the WT, the strains with *Aoran1* deletion showed a remarkable increase in tolerance to low concentrations of NaCl (0.1 M); however, there was no significant difference in tolerance to higher concentrations of NaCl (0.2–0.3 M) or sorbitol (0.25–0.75 M) (Figure S2A,B).

### 3.7. AoRan1 Regulates LD Accumulation and Autophagic Level

**Figure 6:**
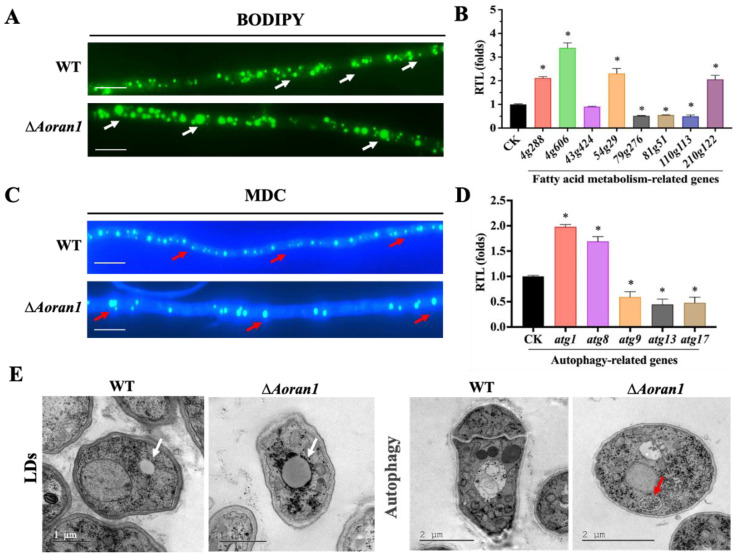
Observation of LD accumulation and autophagy of fungal strains. (**A**,**C**) Microscopic images of LDs and autophagosomes in mycelial cells. LDs in (**A**) and autophagosomes in (**C**) were stained by means of BODIPY dye and MDC staining, respectively. White arrows: LDs. Red arrows: autophagosomes. Bar = 5 μm. (**B**,**D**) Relative transcription level (RTL) of genes related to fatty acid metabolism (**B**) and autophagy (**D**) examined in mutant strain versus WT strain at day 5. CK in (**B**,**D**) was denoted as standard for statistical analysis of relative transcription level of gene in mutant versus WT strain. (**E**) TEM showed internal ultrastructure of hyphae. Red arrow: autophagosomes. White arrows: LDs. Asterisk: Significantly different compared to WT strain (* *p* < 0.05).

BODIPY staining showed a significant increase in LD accumulation in the Δ*Aoran1* mutant strains compared with the WT strain (Figure 6A). The transcription levels of eight genes related to LD metabolism were examined via RT-qPCR, among which the transcription levels of genes encoding short-chain dehydrogenase reductase (AOL_s00004g288), peroxisomal ABC transporter (AOL_s00004g606), peroxisomal hydratase-dehydrogenase-epimerase (AOL_s00054g29), and 3-ketoacyl-CoA ketothiolase (AOL_s00210g122) were significantly upregulated, and the transcription levels of acyl-CoA dehydrogenase (AOL_s00079g276), phosphatidylinositol transfer protein (AOL_s00081g51), and 3-hydroxybutyryl-CoA dehydrogenase (AOL_s00110g113) were considerably downregulated (Figure 6B). MDC staining showed a remarkable decrease in the number but an increase in the volume of autophagosomes (Figure 6C). Among the six autophagy-related genes examined, the transcription levels of *atg1* and *atg8* were significantly upregulated, and the transcription levels of *atg9*, *atg13*, and *atg17* were significantly downregulated (Figure 6D). In addition, the TEM images show more significant LD accumulation and autophagic levels in the Δ*Aoran1* mutant strains than in the WT strain (Figure 6E).

### 3.8. AoRan1 Contributes to Secondary Metabolism

**Figure 7:**
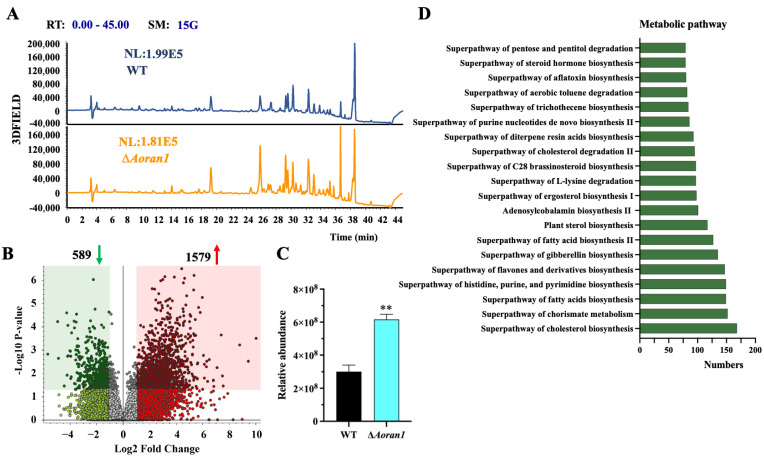
Role of AoRan1 in secondary metabolism. (**A**) Comparison of metabolic profiling between WT and Δ*Aoran1* mutant strains. (**B**) Volcano plot of differential metabolites between WT and Δ*Aoran1* mutant strains. Green arrow represents the number of downregulated compounds, and red arrow represents the number of upregulated compounds. (**C**) Relative abundance of arthrobotrisins between WT and Δ*Aoran1* mutant strains. (**D**) Top 20 metabolic pathways enriched in Δ*Aoran1* mutant strain. Asterisk: Significantly different compared to WT strain (** *p* < 0.01).

The 7-day fermentation broth of the WT and Δ*Aoran1* mutant strains was processed using LC-MS to analyze the differential compounds between them. The chromatograms showed a remarkable increase in the abundance of metabolic compounds in the Δ*Aoran1* mutant strain (Figure 7A). The clustered volcano plot revealed that 1579 compounds were upregulated and 589 compounds were considerably downregulated upon the deletion of *Aoran1* (Figure 7B). Arthrobotrisins, specialized metabolic compounds that can be produced by several NT fungi [43,44], were found in both the WT and Δ*Aoran1* mutant strains (Figure S3A,B), and the relative abundance of arthrobotrisins was significantly increased in the Δ*Aoran1* mutant strain (Figure 7C). The compounds with the most significant differences (top twenty) in the Δ*Aoran1* mutant strain compared to the WT strain are shown in Table S3; among them, the main compounds are tridecyl benzenesulfonate, 16-(3,4-dimethoxybenzylidene), rost-4-ene-3,17-dione, and S_S-dimethyl-beta-propiothetin. The main metabolic pathways associated with these differentially expressed compounds consist of the superpathways of cholesterol biosynthesis, chorismite biosynthesis, fatty acid biosynthesis, and the biosynthesis of histidine, purine, and pyrimidine (Figure 7D).

## 4. Discussion

In recent years, NT fungi have been recognized as efficient and promising nematode control agents [9,45]. The traps produced by NT fungi are crucial tools that enable them to obtain nutrition and also give them a competitive advantage over other non-predatory fungi [5,6], but the regulatory mechanisms associated with trap formation are still not well understood. Therefore, it is important to gain a deeper understanding of how these traps are differentiated, how NT fungi respond to the external environment, and what the relationship is between NT fungi and other organisms [6,46].

The serine/threonine protein kinase Ran1 is a conserved protein from yeast to humans [47]. In this study, *A. oligospora* was used as a model fungus, and the *Aoran1* gene was knocked out using homologous recombination to further characterize its function. After obtaining the mutant strains, we performed a series of phenotypic analyses on them. The results show that the deletion of *Aoran1* did not cause a significant change in the growth rate. However, our results indicate that the resistance of the Δ*Aoran1* mutant strain to cell wall synthesis-disrupting reagents and oxidants was significantly increased compared to that of the WT strain. A previous study demonstrated that in *S. pombe*, the deletion of Ran1 leads to a remarkable impact on the transcriptional induction of cell wall integrity (CWI) MAPK pathway-regulated genes, suggesting that Ran1 may be a regulator of CWI-responsive genes [15], which correlates with our findings. The above results suggest that the role of Ran1 in response to external stress is conserved across fungi.

Studies have shown that for filamentous fungi, conidiogenesis is the most common reproductive strategy for dispersal, invasion, and proliferation in the environment [48]. Previous studies showed that in *S. pombe*, in the presence of mating pheromones, cells will conjugate with the opposite mating type and undergo meiosis and sporulation regardless of nutrient starvation, whereas the role of Ran1 is to prevent the onset of meiosis until conjugation occurs. Therefore, we speculate that Ran1 plays a role in sporulation [49,50]. Here, we found that the knockout of the *Aoran1* gene led to a remarkable increase in conidial production in the mutant strains; they also had significantly higher rates of conidial germination. We also found that *brlA*, one of the key genes controlling the central regulatory pathway of sporulation [51], had a significantly increased transcription level. A previous study showed that the absence of *brlA* leads to a complete loss of the ability to form conidiophores and produce conidia in *A. oligospora* [52]. In *A. fumigatus*, the deletion of *brlA* also caused a complete loss of the ability to produce conidia [53]. These results correlate with our findings, all of which indicate that *brlA* has a crucial role in regulating conidiation. Therefore, AoRan1 is a crucial regulator of conidiation in *A. oligospora*.

As a representative NT fungus, it is interesting to note that *A. oligospora* can form a three-dimensional adhesion network to prey on nematodes [11,13]. The process of trap formation is very complicated and has been proven to be the result of multiple cells or hyphae undergoing fusion [36,54]. Recently, it was proven that the highly conserved Fus3 MAPK of the pheromone response pathway plays a key role in signaling transduction during trap formation [55]. For instance, in *A. oligospora*, the deletion of *Aoham5* and *Aoso* results in the mycelial ring in the trap being incompletely closed, showing a spiral shape. As a result, nematodes still have a chance of escaping after being captured, resulting in a significant reduction in the nematode predation efficiency [36]. Additionally, in *S. pombe*, it was demonstrated that the activation of pheromone-responsive MAPK plays a vital role in inducing cells to undergo phenotypically normal meiosis [56], and it is interesting to note that Ran1 performs a similar function [47]. Therefore, we wondered whether AoRan1 has a regulatory role in trap formation. In this study, we found that the deletion of *Aoran1* caused a remarkable increase in the number of traps at all time points. In addition, the transcription levels of genes encoding serine proteases were remarkably increased in the mutant strains, and all of the above changes may contribute to the remarkable increase in nematode predation ability in the mutant strains. Our results indicate that AoRan1 is crucial for trap formation and proper trap morphogenesis in *A. oligospora*.

A noteworthy point is that autophagy-related pathways participate in regulating various biological processes, such as conidiation, conidial germination, and development, in filamentous fungi [57,58,59]. In this study, we found that the absence of Aoran1 caused a reduction in the number but a significant increase in the volume of autophagosomes in *A. oligospora*. Interestingly, there were also significant changes in the number of conidia and the conidium germination rate in the mutant strain. A previous study suggested that autophagy is crucial in trap formation in *A. oligospora*; it demonstrated that the absence of *Aoatg8* remarkably reduced the autophagic level, disrupted trap formation, and ultimately severely affected the ability of *A. oligospora* to prey on nematodes [38]. In the present study, we observed a remarkably enhanced autophagic level in the mutant strains, and at the same time, the number of traps and the nematode-trapping ability were significantly increased. These results may help to elucidate the relationship between the autophagy process and other biological processes, such as conidiation and trap formation.

Currently, metabolomics has been widely used to illustrate the mechanisms underlying fungal growth and virulence [40,60]. Metabolomics analyses revealed multiple up- and downregulations of secondary metabolic compounds in the mutant strains. In particular, the superpathway of fatty acid biosynthesis was significantly enriched, which is consistent with the results we obtained in relation to fatty acid metabolism. It has been shown that arthrobotrisins are able to inhibit trap formation [44,61,62]. In this study, we found that the relative abundance of arthrobotrisins was significantly increased, which is in contrast to the increased number of traps we found. The most likely explanation is that trap formation is jointly regulated by various biological processes, and the effect of arthrobotrisins on trap formation may be compensated by other biological processes. Therefore, mechanisms related to trap formation in mutant strains still need to be further investigated. In conclusion, AoRan1 is crucial in regulating secondary metabolism in *A. oligospora*.

## 5. Conclusions

This study is the first to characterize the functions of serine/threonine protein kinase Ran1 in NF fungi. Our results reveal that AoRan1 plays crucial roles in various important biological processes, including sporulation, stress response, pathogenicity, and secondary metabolism. In addition, AoRan1 regulates LD accumulation and autophagic levels. These results may expand our understanding of the roles that serine/threonine protein kinase play in *A. oligospora* and lay the foundation for further research on the mechanisms by which Ran1 regulates conidiation, pathogenicity, and other biological processes in NT fungi.

## Data Availability

All data generated or analyzed during this study are included in the published paper and associated Supplementary Files.

## Supplementary Materials

The following supporting information can be downloaded at: https://www.mdpi.com/article/10.3390/microorganisms12091853/s1, Figure S1: Mapping of pRS426 and pCSN44 plasmids used to construct knockout vector for target gene; Figure S2: Comparison of fungal strains in response to osmotic stress reagents; Figure S3: Mass spectra of arthrobotrisins in fungal strains; Table S1: Primers used to knock out the target gene; Table S2: Primers for RT-qPCR detection of genes related to sporulation, fatty acid metabolism, and autophagy in *A. oligospora*; Table S3: The compounds with the most significant differences (top twenty) in Δ*Aoran1* mutant strain.
