# Supplementary material for: AoRan1 Is Involved in Regulating Conidiation, Stress Resistance, Secondary Metabolism, and Pathogenicity in Arthrobotrys oligospora
Source: Microorganisms. 2024 Sep 6;12(9):1853. doi: 10.3390/microorganisms12091853 (PMC11434409; doi:10.3390/microorganisms12091853)
Supplement: Supplementary file 1 [file microorganisms-12-01853-s001.zip › microorganisms-3176469-supplementary.pdf]

Supplementary Materials

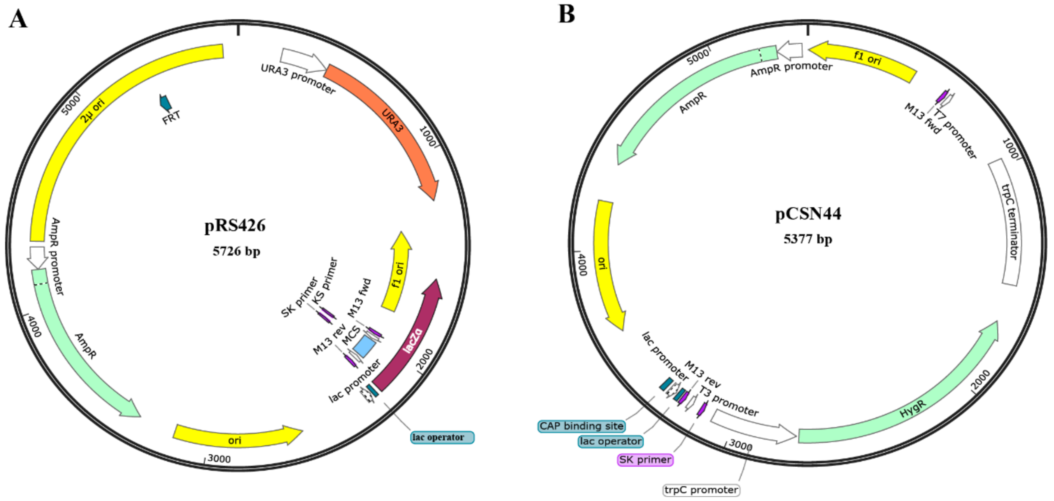

Figure S1. Mapping of pRS426 and pCSN44 plasmids used to construct knockout vector for target gene.

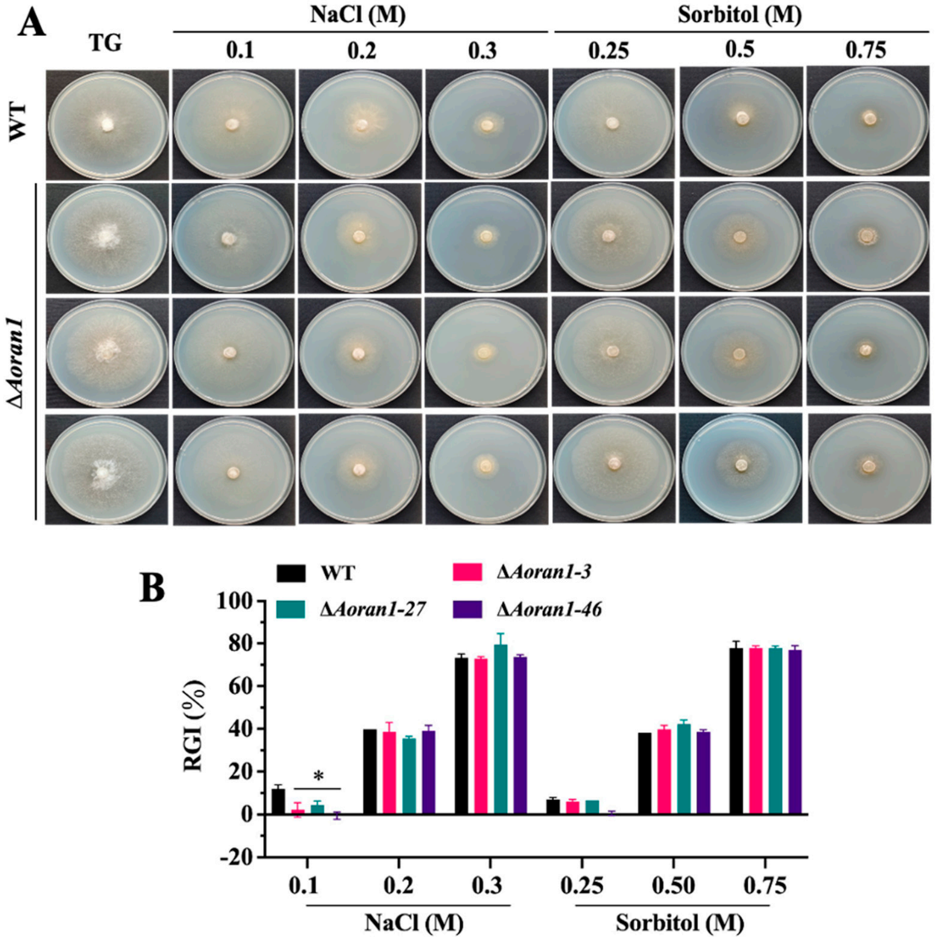

Figure S2. Comparison of fungal strains in response to osmotic stress reagents. (A) Colonial morphology of fungal strains under different concentration gradients of osmotic stress reagents. (B) Relative growth inhibition (RGI) of fungal colonies after 5-day incubation at 28°C on TG plates treated with indicated concentrations of NaCl and Sorbitol. The asterisk indicates a significant difference between WT and Δ*Aoran1* mutant strains (Tukey's HSD, $*p < 0.05$).

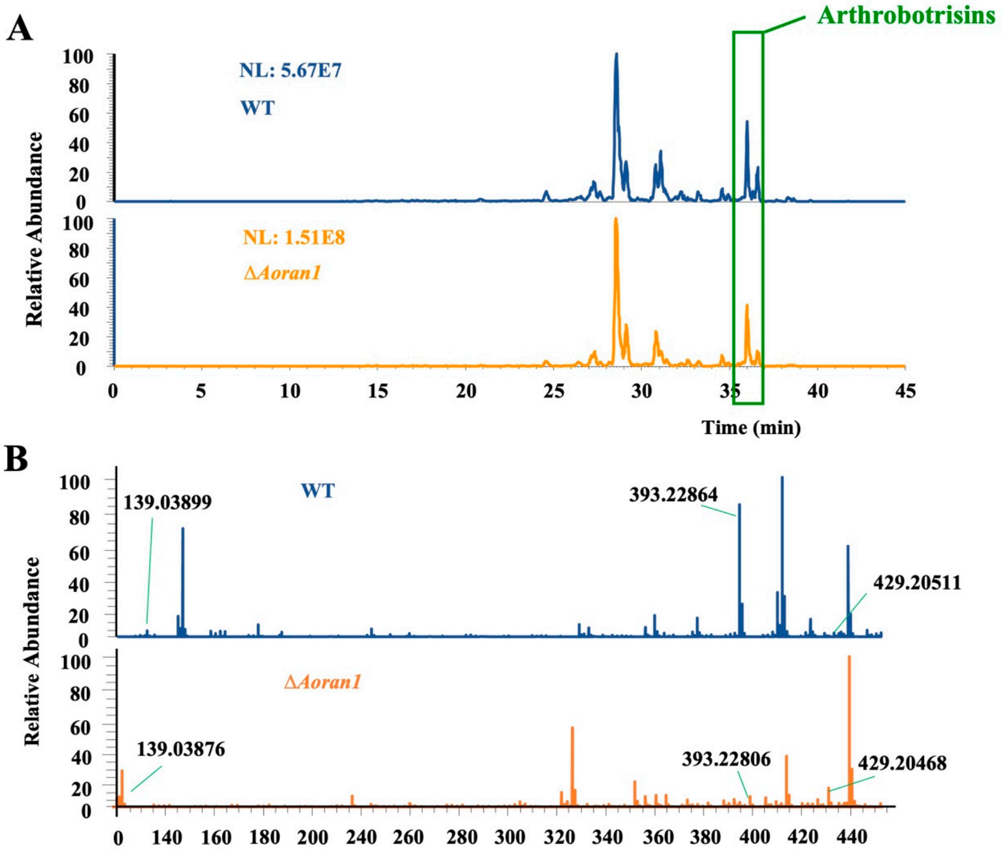

Figure S3. Mass spectra of arthrobotrisins in fungal strains (diagnostic fragment ions at m/z 139, 393, and 429).

Table S1. Primers used to knock out the target gene in this study.

Primer	Sequence (5'-3')
**Amplify *hph* resistance gene**	
HPH-F	GTCGGAGACAGAAGATGATATTGAAGGAGC
HPH-R	GTTGGAGATTTCAGTAACGTTAAGTGGAT
**Amplify the 5' flank of *Aoran1* gene**	
AoRan1-5F	GTAACGCCAGGGTTTTCCCAGTCACGACGACTAACTTTCCGCTTGTG
AoRan1-5R	ATCCACTTAACGTTACTGAAATCTCCAACAATACATCGTCGCACAAC
**Amplify the 3' flank of *Aoran1* gene**	
AoRan1-3F	CTCCTTCAATATCATCTTCTGTCTCCGACCTGGGAGGGTAACGAGAT
AoRan1-3R	GCGGATAACAATTTCACACAGGAAACAGCAAAGCAGGCATAAGGATT
**Verify the transformants**	
YZ-*AoRan1*-F	GCTCCAGTGGTATCAAGTT
YZ-*AoRan1*-R	GCAAGTTCTCATTCGCTAC
**Verify the transformants by RT-qPCR**	
RT-*AoRan1*-F	GGATCTGCTTGTTATGCTTCTG
RT-*AoRan1*-R	CTTCAAGAAAGCTCGGAAAGTG
RT-tubulin-F	CCACCTTCGTCGGTAACTC
RT-tubulin-R	TCGTCCATACCCTCACCAG

Table S2. Primers for RT-qPCR detection of genes related to sporulation, fatty acid metabolism, and autophagy in *A. oligospora*.

Gene name	Sequence (5'-3')	Sequence (3'-5')
**Sporulation-related genes**		
AOL_s00083g25 (*stuA*)	AGCTCCCGAAACGAGTCTAA	ATTGATCATGTGATTATCCT
AOL_s00080g63 (*abaA*)	AACTTTATGCGCCTTGTCGT	TTGGCTAGGTGGTCTGTACG
AOL_s00210g120 (*medA*)	TCCGGCCCAATGATTCAGAA	AGATCGCAGGAACATGGTGA
AOL_s00083g487 (*lreA*)	TTCTCTTCGTCCCAAGCCAC	ACCGGTTCGAGTGGAGTCTA
AOL_s00080g93 (*lreB*)	CCAGGGTCGTCAGTATCTT	CAGCATCTTCCAGGTCAA
AOL_s00007g157 (*flbC*)	CTCTCCGGCAAAGACAATCG	GTCGACTGAGGATAGTAGCT
AOL_s00173g221 (*wetA*)	TTACATGCCACCCCAAGTCC	CAATTGCAACTGCGTCCACA
AOL_s00097g514 (*brlA*)	TTGAGGCCTCGATCCGTAGA	AGGTAGATGGCGCTGTTACG
**Fatty acid degradation related genes**		
AOL_s00004g288	AAGAAATCCCACTTCAGAGAGG	TACGTGTCCAGTAACATAGCTC
AOL_s00004g606	TTCGGATTCGTTATTACCTCCC	TAACATGAGTCGCTTGTTTGTG
AOL_s00043g424	CTCTGCTCTATGGATACGAACA	AGATGAACTTCTCGACTTCTCC
AOL_s00054g29	GGTATCTACGGAAATTTTGGCC	GTGCAATATAATCGGGCTTGAG
AOL_s00079g276	AACAATCCGTCGTTATTGTTCC	GCGATCATGTAGTCTAGTCCTC
AOL_s00081g51	GCCGATCCTTACCAAATCATTC	CCAATTCTTTTCCGTAGCTGAG
AOL_s00110g113	CTAACAGAAACTCAAGCATCGG	GGAACCGGATTCATGAAATGAG
**Autophagy-related genes**		
AOL_s00076g234 (*atg1*)	GCCATTAGATTTGCCACCAG	CAGTTCCGTTCGTCACTCCC
AOL_s00007g534 (*atg8*)	AGCGTATCAAGCTGTCTCCC	CTCGTAGCCGAAGGTGTTTT
AOL_s00054g371 (*atg9*)	TTCTTCGGAGGACAGAGCAT	CTAGGTTTGCGTGAAAGTCG
AOL_s00215g74 (*atg13*)	AGAGGTGGAGGGTTGATTTA	GGAGTTTCCATGATGGCAGT
AOL_s00043g575 (*atg17*)	GAGATTCAGAACTCGTGGTT	AAGAAGTGAGGGTGTATTGC

Table S3. The compounds with the most significant differences (top twenty) in Δ*Aoran1* mutant strain.

Name	Formula	MW	RT (min)	log2 Fold change	P-value
Tridecyl benzenesulfonate	$C_{19}H_{32}O_3S$	340.20747	38.685	10.05	0.06590238
Ceramide ap	$C_{36}H_{73}NO_5$	599.5484	38.709	6.06	0.001754039
16-(3,4-Dimethoxybenzylidene) androst-4-ene-3,17-dione	$C_{28}H_{34}O_4$	434.2437	37.741	7.49	0.04156306
Terikalant	$C_{24}H_{31}NO_3$	381.23104	34.223	6.12	0.02504023
UNII:1JCX24ZXMH	$C_{26}H_{36}N_4O_4$	468.27357	38.22	3.5	0.012284502
S_S-Dimethyl-beta-propiothetin	$C_5H_{10}O_2S$	134.04014	4.21	6.4	0.004295312
Benzyl(2R_3S)-2-methyl-3-hydroxybutanoate	$C_{12}H_{16}O_3$	208.11007	37.904	4.39	0.009148995
1,4-Bis({3-[(2-hydroxyethyl)amino]propyl}amino)-9,10-anthraquinone	$C_{24}H_{32}N_4O_4$	440.24151	18.35	4.37	0.01906226
Eugenolmethylether	$C_{11}H_{14}O_2$	178.09957	38.735	3.74	0.03890032
2-Hydroxyethyl benzenesulfonate	$C_8H_{10}O_4S$	202.02946	31.996	-2.97	0.003887421
2,3-Dihydroxyhexacosanoic acid	$C_{26}H_{52}O_4$	428.38657	42.221	-4.87	0.0022815
2-Methyl-6-oxohepta-2_4-dienal	$C_8H_{12}O_2$	140.08383	22.864	4.53	0.03273206
2-Dodecylbenzenesulfonic acid	$C_{18}H_{30}O_3S$	326.19195	35.102	3.71	0.000374958
N-[2-(4-tert-butylphenoxy)ethyl]-5-(4-methylpiperazin-1-yl)-2-nitroaniline	$C_{23}H_{32}N_4O_3$	412.24651	28.855	4.54	0.013428662
Butyl cyclohexyl phthalate	$C_{18}H_{24}O_4$	304.1678	29.814	4.09	0.03038478
Mucronine A	$C_{29}H_{38}N_4O_4$	506.28844	31.302	4.32	0.02838919
Docosanoic acid methyl ester	$C_{23}H_{46}O_2$	354.35021	38.636	4.93	0.009760723
dilauroyl peroxide	$C_{24}H_{46}O_4$	398.33984	38.651	4.79	0.037938972
Norethisterone acetate	$C_{22}H_{28}O_3$	340.20413	37.216	-4.25	0.00163836

[SThydrox]3beta_16alpha-dihydroxy-5-androsten-17-one	$C_{19}H_{28}O_3$	304.20416	39.265	-3.88	0.000185283
